# Survey of Academic Field Experiences (SAFE): Trainees Report Harassment and Assault

**DOI:** 10.1371/journal.pone.0102172

**Published:** 2014-07-16

**Authors:** Kathryn B. H. Clancy, Robin G. Nelson, Julienne N. Rutherford, Katie Hinde

**Affiliations:** 1 University of Illinois, Urbana-Champaign, Department of Anthropology, Urbana, Illinois, United States of America; 2 Skidmore College, Department of Anthropology, Saratoga Springs, New York, United States of America; 3 University of Illinois, Chicago, Department of Women, Children, and Family Health Science, Chicago, Illinois, United States of America; 4 Harvard University, Department of Human Evolutionary Biology, Cambridge, Massachusetts, United States of America; University of Pennsylvania, United States of America

## Abstract

Little is known about the climate of the scientific fieldwork setting as it relates to gendered experiences, sexual harassment, and sexual assault. We conducted an internet-based survey of field scientists (N = 666) to characterize these experiences. Codes of conduct and sexual harassment policies were not regularly encountered by respondents, while harassment and assault were commonly experienced by respondents during trainee career stages. Women trainees were the primary targets; their perpetrators were predominantly senior to them professionally within the research team. Male trainees were more often targeted by their peers at the research site. Few respondents were aware of mechanisms to report incidents; most who did report were unsatisfied with the outcome. These findings suggest that policies emphasizing safety, inclusivity, and collegiality have the potential to improve field experiences of a diversity of researchers, especially during early career stages. These include better awareness of mechanisms for direct and oblique reporting of harassment and assault and, the implementation of productive response mechanisms when such behaviors are reported. Principal investigators are particularly well positioned to influence workplace culture at their field sites.

## Introduction

For many social, life, and earth science disciplines, conducting research in field settings is an integral component of scholarship. The ability to explore various ecological and cultural settings attracts many young researchers to their respective disciplines. Many university science programs require fieldwork for both undergraduate and graduate degree completion [1], [2]. Additionally, researchers in field-based sciences with active research sites have been shown to write more papers and secure more grants than those without them [3]. Thus, a non-trivial amount of research in the sciences is generated in the field context.

As an important component of professional training and scholarship, substantial preparation for fieldwork is essential at individual, laboratory, and institutional levels. Fieldwork preparation includes coordinated efforts in project design, oversight approval of protocols (i.e. IRB, IACUC), grant submission and funds management, logistical practicalities, and “boots on the ground” research activities. Faculty, however, are rarely trained in the interpersonal skills of conflict management, negotiation, and resolution that would allow them to informally and formally confront personnel issues as they arise and before they can escalate [4], [5]. Prioritization of data-generation has the potential to contribute to the neglect - benign or otherwise - of team dynamics such that alienation, harassment, and assault may occur and thereby diminish scientists' field experiences.

Workplace climate has been investigated across many professional settings [6], [7], [8], [9], including the professorate [10], [11], [12]. In particular, sexual harassment and assault have received considerable attention. Sex discrimination harassment is the harassment of a person because of their sex; however, defining sex-based harassment poses challenges because of differential subjective experiences of the same phenomena [13], [14], [15], [16]. According to the United States Equal Employment Opportunity Commission [17], sexual harassment includes not only unwelcome sexual advances, but also offensive remarks about a person's sex. While the legal definition of sexual assault varies by state across the United States of America, at its most basic, the term refers to any unwanted sexual contact, up to and including rape. While male to female harassment and assault are the most common, incidents can occur between individuals of the same sex, and females can harass or assault males [14].

A hostile work environment is not only harmful to productivity and psychological well-being, but reduces job satisfaction and increases job turnover [18], [19]. This area of organizational and behavioral research, however, has not broadly surveyed scientists about their experiences while engaged in professional fieldwork. In order to better understand the diversity of experiences in field research settings, particularly sexual harassment and sexual assault, we conducted an online survey (N = 666 respondents), the first wave of which targeted biological anthropologists (N = 124), and the second, field scientists more broadly (N = 542). Our study investigated three key questions: 1) do respondents experience harassment and assault at field sites? If so, 2) who are the targets and perpetrators of harassment and assault? And 3) do field sites have codes of conduct and effective reporting mechanisms available to targets of abuse?

## Methods

### Ethics statement

We obtained human subjects approval from the University of Illinois Institutional Review Board (#13520). Informed consent was obtained from all respondents. As the research measure was an online survey, the front page text informed potential respondents about the study, and that continuing on to the survey signified consent to participate.

### Study Construction

The survey was designed to generate information about respondents' fieldwork history (i.e. number of field sites, field site management structures) and then in greater detail about their most recent or most notable field experience. The survey used operationalized definitions of phenomena generally characterized as “harassment” by the United States Equal Employment Opportunity Commission [17], and “assault” by WomensLaw.org [20] without specifically using the terms “harassment” or “assault” to avoid making respondents name their experiences (see Materials S1 for the full survey). This design is consistent with other studies [21] that address prevalence of these phenomena because the survey data allow for objective and subjective assessments of experience. Further, the target is not the only one to experience harassment and assault, as bystanders may also observe and be influenced by witnessing it. Targets of harassment and assault are sometimes labeled as “sensitive,” or over-reactive, by critics of the severity of the effects of sexual harassment [22]. Thus, multiple perspectives contribute to the climate of a field site [21].

The survey included 45 questions (44 for the first wave, as the second wave added one question about the respondent's discipline). For each question in the survey, respondents could decline to answer, and sample size for each question is provided when presented in the results. No questions were asked about specific field sites, locations, team size, or dates of study to maximize privacy protections for respondents. The survey questions were distributed among several categories: 10 demographic questions; 17 questions on general field site work environment, which ranged from questions about the gender ratio at the respondent's field site, to the principal investigator's gender, to the presence or absence of sexual harassment policies, or codes of conduct; and 18 questions on sexual harassment and assault (Materials S1).

This last set queried about both observed and direct experiences, as well as situation outcomes when the respondent reported personal harassment or assault. Respondents were also able to provide a free response to the question “*With what frequency did you observe or hear about other field site researchers and colleagues making inappropriate or sexual remarks*?” No examples or prompts were provided in concert or in the questions prior to this question. The following questions could be answered as “Yes,” “No,” or “I don't know:”

*“Have you ever personally experienced inappropriate or sexual remarks, comments about physical beauty, cognitive sex differences, or other jokes, at a field site? (If you have had more than one experience, the most notable to you).”*

*“Have you ever experienced physical sexual harassment, unwanted sexual contact, or sexual contact in which you could not or did not give consent or felt it would be unsafe to fight back or not give your consent at a field site? (If you have had more than one experience, the most notable to you).”*

Respondents were asked whether there were mechanisms in place to report if they experienced unwanted comments or physical contact, and if so, whether they reported the most notable incident they experienced. Respondents could additionally indicate whether they were satisfied with the response if they did report the incident. Respondents could also describe the mechanisms for reporting in a free-write box included on the survey. These data will be described in a forthcoming manuscript.

### Survey Recruitment

Researchers distributed the link to the survey to potential respondents through e-mail and online social networks (Facebook, Twitter, and LinkedIn). Links to the survey on field experiences were posted on Facebook group pages for the Evolutionary Anthropology Society Social Network, Biological Anthropology Developing Investigators Troop, Biological Anthropology Section of the American Anthropological Association, Membership of the American Society of Primatologists, and BioAnthropology News. These links were then shared and retweeted by colleagues and disseminated using chain referral sampling (in a snowball manner) [23]. Links to the survey were also provided on science and service blogs operated by two of the study's authors [24], [25], [26] (KC and JR) and at the conclusion of print and online news reports of the ongoing study [27].

The survey was conducted in two waves: the first, aimed at biological anthropologists between February 21^st^ and April 12^th^ 2013 (N = 124); and the second from April 13^th^ to May 10^th^ 2013 (N = 542) that allowed respondents to provide their professional discipline. This addition to the survey was in response to feedback requesting opportunities for other disciplines engaged in field research to participate in the study. Survey respondents could indicate whether they were willing to be contacted for a subsequent, 30-minute phone interview; 26 interviews were completed between the two waves and all were conducted by KC. Interviews were designed to allow respondents to describe their range of experiences at the field sites where they had trained or worked; these data will be described in subsequent publications.

### Statistical Analysis

Descriptive statistics of respondent characteristics and questions answered were generated and examined for errors and extreme values. For questions about number of field sites at which respondents have conducted research, potential answers were 1, 2, 3, and ≥4. Respondents generated a diversity of answers to the free question “*With what frequency did you observe or hear about other field site researchers and colleagues making inappropriate or sexual remarks*?” which were then binned into never, rarely, regularly, and frequently categories (Table 1). KH categorized these answers blind to all respondent characteristics and answers to other questions in the survey. To generate descriptive means these were converted into numerical values truncated at 4. For questions for which answers could be “yes,” “I don't know” or “no,” (e.g., “*Did any of the field sites have a code of conduct*?”), KH and JR conservatively bifurcated responses into “yes” and “not yes.” Chi-square, t-test, and regression models were constructed in JMP 9.0 (SAS, Inc).

**Table 1:** Free-write responses to “With what frequency did you observe of hear about other field site researchers and colleagues making inappropriate or sexual remarks?” categorized into never, rarely, regularly, and frequently.

**Never**: ‘0,’ never, I can't remember a single one, I was never aware of this, I can't think of any, no, none, not at all, zero.
**Rarely**: 1, rare, 3 or more times, a few times, about once a season, almost never, almost none, annually, uncommon, only occasionally, extremely rarely, from time to time, monthly, infrequent, little frequency, on occasion, low, low frequency, mild-low, not frequent, not much, not often, not too much, not very frequent, not very often, occasionally, occurred, once, pretty much never, rare, rarely, relatively infrequently overall, seldom, some frequency, several times, slight sometimes, somewhat, twice, very infrequently, very low, very low frequency, very rarely, very seldom, yes
**Regularly**: every other day, bullying was rampant, moderately frequently, often, 4%, 20%, 10, <5%, 10–15% of the time, 3–4/week, 3–5 times per week, 5% inappropriate remarks, a few times a week, at least once a week, at least weekly, regularly, commonly, often enough it was no longer shocking, every few days, every other day, every second day, fairly common, fairly frequent, very very common, fairly often, often, regular, a lot, commonplace, maybe a few times a week, maybe once or twice a week, moderate, multiple times per week, few times a week, increasingly across the season, once a week, not uncommonly, once a week, pretty frequent, rarely but regularly, regularly, several times a week, frequent-constantly, twice a week, weekly, The late 60 s when I did field work? Are u kidding?, weekly, with some frequency
**Frequently**: 40%. 50%, 60–75% of the time, a lot, all of the time, almost constantly, almost daily, almost every day, at every meeting, daily, constantly, continuously, every day, extremely frequent, frequently, high, many times per day, too often, most of the time, often, quite a bit, quite frequently, several times each day, very frequently, very high, very often

### Study Limitations

The data presented here represent the first systematic investigation of field site work environment and experiences, particularly as they relate to sexual harassment and assault. These data are limited in several important ways. First, incivility, chilly climate, sexual harassment, and sexual assault are biopsychologically intense experiences for the targets, witnesses/bystanders, and perpetrators. Recall of these experiences has the potential to precipitate emotional distress. The sample was potentially biased by ethical, pre-participation disclosure that questions regarding these topics were in the survey. Some people may have been more likely to participate in the survey if they had negative experiences, some people may have been more likely to forward the survey link to individuals who had previously disclosed negative experiences in private conversation (snowball sampling), and some people may have been less inclined to participate in this survey to avoid emotional stress of sharing their experiences. Several colleagues directly informed the study authors that they would not participate because revisiting their experiences was too traumatic. Thus, it is unclear if the self-selection of this sample produces over- or under-reporting of negative field experiences.

One potential concern one could have was that individuals with negative experiences could take the survey multiple times, becoming disproportionately represented in the dataset of their experiences. However, nearly all respondents provided a unique identifier in the form of an e-mail address (N = 628, 94.3%). Comparison between the group that provided a unique identifier and those that did not (N = 38) revealed that the two groups did not significantly differ in the composition of their gender, sexual orientations, race/ethnicity, ages, countries of origin, or career stages (all p>0.4). We combined the two groups for subsequent analyses, but did evaluate for differences in harassment and assault (see results).

Although we have substantial confidence that each participant is unique in the dataset, multiple individuals may have worked at the same field site. In the interest of anonymity, the survey did not include questions about specific field site locations preventing nesting these data in regression analyses. As such, these survey data neither allow us to estimate the rate of these experiences among our trainees and colleagues, nor do they allow any estimation of the prevalence of field sites with a hostile work environment and/or systematic abuse. That said, the large number of respondents from across dozens of disciplines and high prevalence of harassment and assaults suggests that the results presented here are likely not attributable to only a handful of hostile field sites. Some field sites represent multi-institutional and international collaborations with researchers from a diversity of cultures, disciplines, and laboratories. Such arrangements have complex and, at times, delicate management dynamics, which were not evaluated in the present study.

## Results

### Respondent Demographics and Field Site Structure

Hundreds of respondents, recruited online, answered our survey questions. A majority of the sample were women N = 516/666 (77.5%). To protect individual respondent identity, we report the majority of respondents for each categorical attribute. We do not report precise categorical descriptions, as some descriptors are distinct and, occasionally, entirely unique, jeopardizing anonymity. Collectively, respondents identified as six different sexual orientations, although the majority identified as heterosexual (N = 572/666, 85.9%). Eight respondents declined to state gender or designated gender other than male or female, these eight individuals are excluded from analyses comparing men and women. Respondents represented a diversity of racial identities, however N = 581/666 (87.2%) identified solely as Caucasian. The participation of minorities in our survey was low, due in part to their under-representation in the Life and Earth Sciences in the United States [28]. Indeed, a majority of respondents were from the United States (N = 498/666, 74.8%), although respondents originated from 30 countries. Respondents (N = 628/666) identified themselves as undergraduate and graduate students, postdoctoral scholars, non-tenure track faculty, tenure track faculty, tenured faculty, emeritus faculty, retired faculty, employed in research, and individuals that did not identify with being in academic positions. Students and postdocs were binned into “Trainees” (N = 386/666, 58%). Adjunct, tenure-track, and tenured faculty were binned into a “Faculty” category (N = 179/666, 26.9%), though there are notable power differentials among these groups. Employees (N = 20, 3%) and Non-Academics (N = 43, 6.5%) were the last two categories. Women and men were represented among these professional categories in proportion to the full dataset. Women outnumber men in undergraduate and graduate study in most field-based sciences [29]; within our sample, however, women were overrepresented. The greater response rate of women may not be atypical for such surveys [10]. Collectively, respondents were from 32 different disciplines across the life, physical, and social sciences. Nearly half of the study participants self-identified as anthropologists from several subfields (applied, biological, linguistic, medical, physical, psychological, and socio-cultural) (N = 319/666, 47.9%). Nearly a quarter of the sample self-identified as archaeologists (N = 159, 23.9%). The rest of the sample comprised biologists (N = 68, 10.2%); zoologists (N = 31, 4.7%); geologists (N = 29, 4.4%); other life, environmental, and agricultural scientists (N = 22, 3.3%); and other social scientists (N = 12, 1.8%). Over 50% of respondents in the survey had conducted research at four or more field sites (N = 365/666, 54.8%), with no difference between men and women's reporting in number of field sites (3.3±0.08 vs. 3.1±0.05, X^2^ = 2.96, df = 3, p = 0.40, N = 658). Respondents had worked at an average of 3.2±0.04 field sites, however, on average, respondents had only ever worked at one field site directed by a woman (1.3±0.04).

### Do Sexual Harassment and Assault Happen at Research Field Sites?

**Figure 1:**
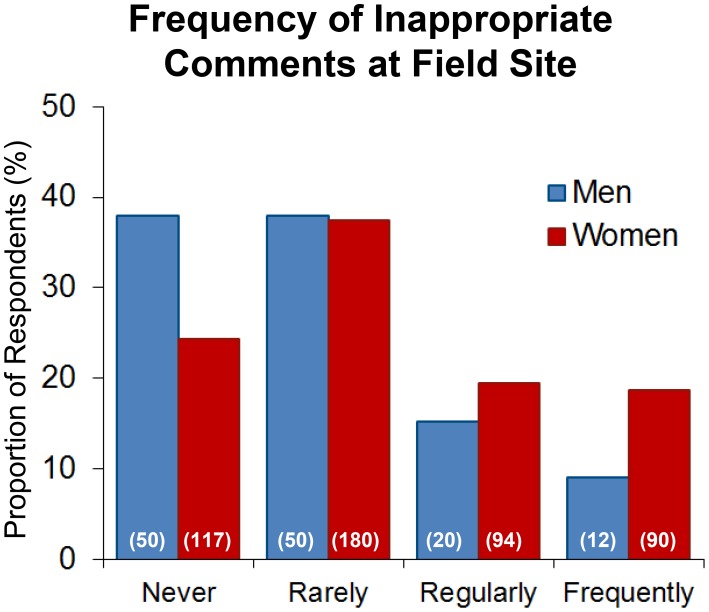
Proportion of survey respondents, by gender, who indicated that inappropriate or sexual comments occurred never, rarely, regularly, or frequently at their most recent or most notable field site (N).

A majority of survey respondents reported that they had directly observed or been told about the occurrence of other field site researchers and/or colleagues making inappropriate or sexual remarks at their most recent or most notable field site (N = 448/619, 72.4%). Men and women, however, characterized the frequency of such comments slightly differently, with men skewing lower in frequency than did women (Figure 1). Men were more likely to report that comments never occurred, whereas women were more likely to report that comments occurred frequently (X^2^ = 14.2, p = 0.003, df = 3, N = 613). Recoding these categories numerically (0 = Never, 1 = Rarely, 2 = Regularly, 3 = Frequently) allowed us to calculate an operationalized mean for work environment and compare among respondent categories. As expected from the Chi-square analysis, mean values differed between men and women (1.95±.09 vs. 2.33±0.05, t = 3.71, p = 0.0002) although gender of respondent only accounted for ∼2% of the variance in reporting of the general frequency of such comments (R^2^ = 0.022).

A majority (64%, N = 423/658) of all survey respondents, stated that they had personally experienced sexual harassment: i.e. inappropriate or sexual remarks, comments about physical beauty, cognitive sex differences, or other such jokes. Over 20% of respondents reported that they had personally experienced sexual assault: i.e. physical sexual harassment, unwanted sexual contact, or sexual contact in which they could not or did not give consent, or felt it would be unsafe to fight back or not give consent (N = 140/644, 21.7%). Respondents who declined to provide a unique identifier were *less* likely to have experienced sexual harassment than were respondents who provided a unique identifier (18/37 vs. 405/621, X^2^ = 4.0, p = 0.05, df = 1, N = 658), but there was no difference in their experience of sexual assault (8/37 vs. 132/607, X^2^ = 0.003, p = 0.99, df = 1, N = 644).

### Who are the Targets and Perpetrators?

Among survey respondents, harassment and assault at field sites were overwhelmingly aimed at trainees (students at all stages and postdocs) and employees (Table 2). Over 90% of women and 70% of men were trainees or employees at the time that they were targeted; 5 of the trainees who reported harassment were in high school at the time of the incident. Gender was a significant predictor of having personally experienced sexual harassment, with women respondents 3.5 times more likely to report having experienced sexual harassment than men (70% of women (N = 361/512) and 40% of men (N = 56/138), X^2^ = 40.8, p = 0.0001, df = 1, OR = 3.5, N = 650). Women were significantly more likely to have experienced sexual assault: 26% of women (N = 131/504) vs. 6% of men (N = 8/133) in our sample (X^2^ = 30.3, p = 0.0001, df = 1, OR = 5.5, N = 637).

**Table 2:** Distribution of survey respondents who experienced inappropriate comments (harassment) or unwanted physical contact (assault) by gender and professional status at the time of the event.

			Respondent's Status at Time of Experience*		
Experienced	Gender	All	Trainee	Employee	Faculty
		% (N)	% (N)	% (N)	% (N)
**Harassment**	Women	71% (361/512)	84% (305)	12% (42)	2% (8)
	Men	41% (56/138)	68% (38)	20% (11)	13% (7)
**Assault**	Women	26% (131/504)	86% (113)	11% (14)	2% (3)
	Men	6% (8/133)	75% (6)	0% (0)	25% (2)

*Not all respondents provided an answer to these questions.

**Figure 2:**
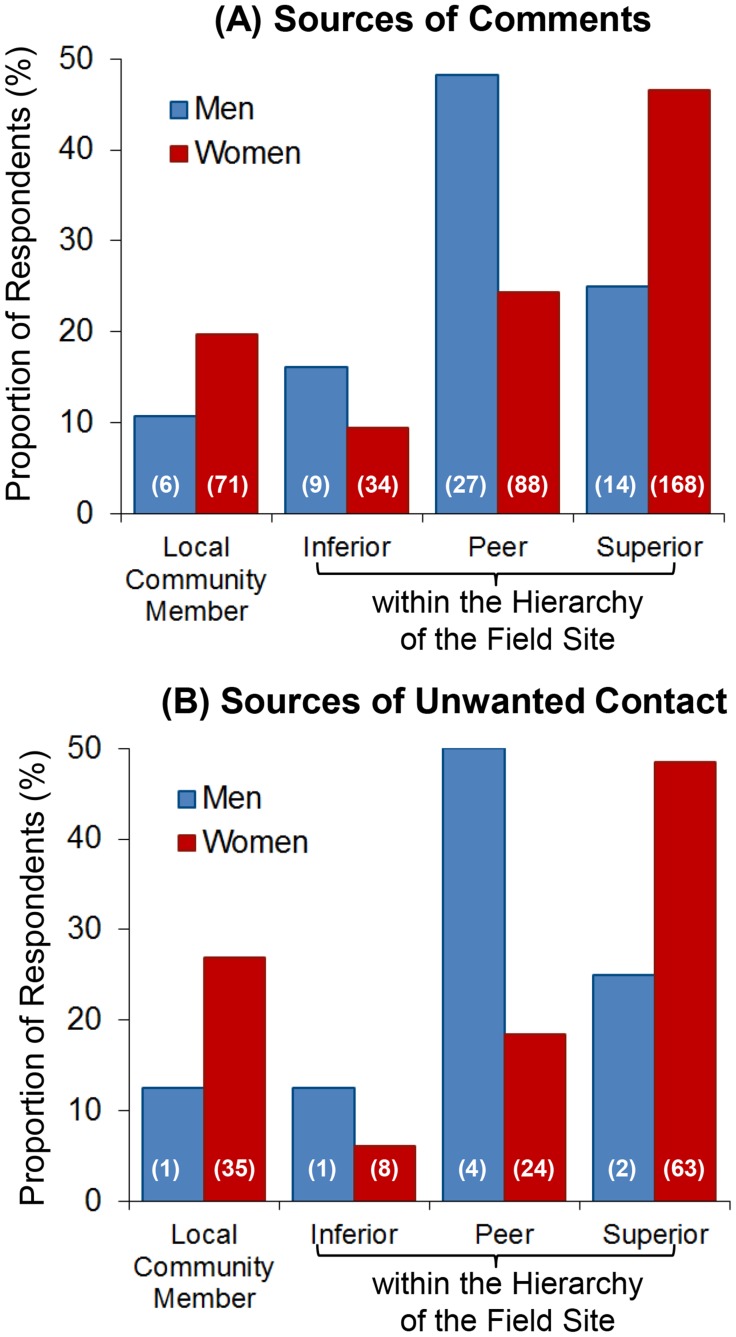
Sources of Harassment (A) and Assault (B) for men and women respondents.

The perpetrators of harassment and assault differed between men and women. Harassment aimed at men primarily originated from peers at the field site (horizontal dynamics) whereas they originated from superiors when directed toward women (vertical dynamics) (X^2^ = 18.7, p = 0.0003, df = 3, N = 417, Figure 2A). Similar patterns were evident for sexual assault. Such behaviors aimed at men originated primarily from peers, whereas such behaviors aimed at women primarily originated from individuals the respondent identified as superior to them in the field site professional hierarchy (Figure 2B). Statistical testing was limited by cell underpopulation among male respondents. According to our respondents, individuals from the local community were responsible for a minority of cases (Figure 2).

### Are Codes of Conduct and Reporting Mechanisms Prevalent at Field Sites?

**Figure 3:**
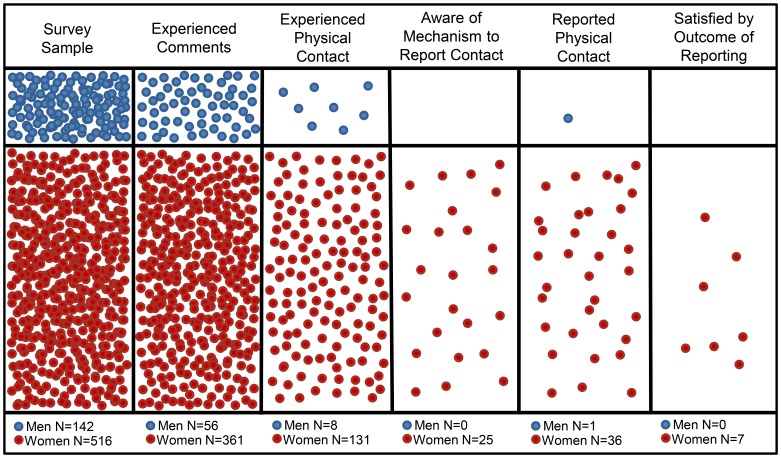
Visual representation of respondents to the survey, their experiences, and who were aware of, made use of, and were satisfied by mechanisms to report unwanted physical contact. Each circle represents one survey respondent. Area for men and women is representative of their relative proportion of survey respondents. Eight respondents declined to provide a dichotomous gender designation and are not represented on this graph.

Respondents typically had limited awareness of workplace policies or mechanisms for reporting. Fewer than half of survey respondents recalled ever encountering a code of conduct at any of the field sites at which they had worked (N = 251/666, 37.7%). Fewer than one fourth of respondents recalled having ever worked at a field site with a sexual harassment policy (148/666, 22.2%). Men were significantly more likely to report having ever worked at a field site that had code of conduct (46.1% vs. 36.4%, X^2^ = 4.36, p = 0.037, df = 1, N = 644) and/or a sexual harassment policy (30.2% vs. 20.0%, X^2^ = 6.39, p = 0.012, df = 1, N = 651) than were women. Study participants who had experienced harassment or assault were also asked about reporting mechanisms and outcomes of reporting. Of those who responded to this particular set of questions, about 20% (N = 87/422; N = 70/360 women and N = 17/56 men) indicated that they were aware of a mechanism to easily report being harassed at the time. Of respondents who experienced assault and answered the survey question, 18% said that yes they were aware of a mechanism to report assault (N = 25/138; N = 25/130 women and N = 0/8 men). Some respondents did report their harassment and assault, including some who did not indicate that they knew an official mechanism by which to do so. Among survey participants, N = 67 women and N = 11 men reported being harassed; N = 36 women and N = 1 man reported being assaulted. Only 18% of respondents who reported harassment were satisfied by the outcome of their reporting (N = 14/78). Over half described themselves as being dissatisfied or very dissatisfied with the outcome of reporting harassment (N = 38/67 women and N = 6/11 men). Only 19% of respondents who reported assault were satisfied by the outcome of their reporting (N = 7/37). Nearly 3/4ths described themselves as being dissatisfied or very dissatisfied with the outcome of reporting assault (N = 25/36 women and N = 1/1 man). (Figure 3).

## Discussion

### Field Sites as Workplaces

Our survey revealed that conducting research in the field exposes scientists to a number of negative experiences as targets and as bystanders. The experiences described by our respondents ranged from inadvertent alienating behavior, to unwanted verbal and physical sexual advances, to, most troublingly, sexual assault including rape. These proportions of respondents experiencing harassment are generally consistent with other studies of workplace harassment in other professional settings [14], [16]. Although men and women at all career stages were exposed to or targeted for harassment and assault, trainee women were disproportionately more likely to report such experiences. Similarly, in a sample of medical trainees, 22% of males and 73% percent of females had experienced workplace sexual harassment during their residency [30]. Moreover the experiences of women most often occurred in the context of power differentials; half of such experiences originated from individuals senior to the target in the professional hierarchy of the research team. In contrast, those men in our sample also targeted for harassment and assault most often experienced inappropriate comments or unwanted contact originating from peers. Conventional wisdom often attributes the majority of sexual misconduct to locals and cultural differences, an important consideration for, for instance, the international business workplace [15]. Incidents perpetrated by locals certainly exist and are traumatic [31], [32], but represented a small minority of cases in our survey. Although women in our sample observed or heard about inappropriate comments more than did men, we are not able to determine if this difference reflects disparity in experiences [14], [16] or differences in perception [33], [34], [35], [36], as both may be operating in the workplace.

The experience of workplace aggression is a serious stressor for victims, negatively affecting not only job satisfaction and performance, but also psychological and physical health [37], [38], [39]. A recent meta-analysis of over 50 studies of workplace aggression found that the specific relationship between the perpetrator and victim was a significant mediator of those negative outcomes [38]. When the perpetrator was a supervisor, targets reported significantly more impaired job satisfaction and commitment, and greater psychological distress, compared to when the perpetrator was a co-worker or an organizational outsider such as a patient or client. Similarly, Chan and colleagues in their meta-analysis incorporating results from nearly 90,000 subjects not only replicated the negative work and health outcomes for targets of workplace sexual harassment, but determined that these negative effects were greater when the target was younger [39]. In our study, respondents were most likely to indicate that experiences of sexual harassment and sexual assault occurred when they were trainees. This suggests that the experience of harassment or assault during the early career stage may have the most negative impact on the most professionally vulnerable in our disciplines. Moreover, bystanders to workplace incivility, particularly women, are demoralized even though they are not the direct targets of the perpetrator [36].

Framing sexual harassment and assault as workplace aggression, this suggests that women may experience greater reductions in satisfaction and commitment to work being conducted in the field (with potential ripple effects to their experience in the entirety of professional science) as well as greater psychological harms than those experienced by men. Barling suggests that workplace aggression may limit victims' cognitive and emotional reserves, leaving them less energy to devote to job performance [40]. Poorer job performance itself may lead to even more targeted aggression, creating a powerful cycle of disadvantage. Given that a much greater proportion of women than men in our survey, as in other studies, reported being targets of sexual harassment or assault [41], these negative experiences may represent a major drain on professional effectiveness, thus contributing to the higher attrition rates of women in the sciences [39], [42]. It must be emphasized that men were also targets of harassment and assault in our study. However, these forms of workplace aggression occurred via mostly horizontal rather than vertical channels, suggesting that the impacts on job performance and psychological well-being are not totally comparable in quality and quantity to those experienced by women.

Experiences of harassment and assault not only have substantial impact on the individual professionally and personally [38], [43], they can also influence the entire scientific community. Social scientists posit that demographic diversity enhances innovation, creativity, and team performance and productivity [44], [45], key aspects of scientific research. Women trainees have outnumbered male trainees across many field-based sciences for more than a decade, however women continue to be under- represented within the professorate [29]. This study joins a growing body of literature documenting the systemic challenges that women scientists confront throughout their careers, challenges that in turn have an effect on the production of science. Among science, technology, engineering, math and medicine (STEM) fields, women are rated as less competent and offered less mentoring [46], are less often included in symposia organized by men [47], and as faculty are engaged for fewer conversations about research than are men [48]. Although not yet investigated in STEM publications, women are cited less often in scholarly social science publications [49], [50]. Our results cannot adequately speak to the experiences of people of color or lesbian, gay, bisexual, transgender, or queer/questioning (LGBTQ) individuals because they are under-represented in our fields and therefore our dataset, but the experiences reported by our respondents are likely reflective of a broader climate for members of various minority groups. Field-based science is potentially impoverished by the extent to which hostile field environments contribute to the under-representation of diverse populations at all professional stages. The lack of diverse backgrounds and perspectives may well constrain the range of research topics being addressed, slowing advances and achievements in science.

### Moving Forward

The study represents an important first step in recognizing that sexual harassment and assault occur during scientific field work. Given the retrospective, snowball sampling methodology, our study is not able to determine the prevalence of these negative experiences within or across disciplines, nor those that occur in the classroom, laboratory, or at professional conferences. However, it does reveal a systemic and substantial degree of problematic behavior within scientific disciplines. Despite the expectation that codes of conduct, principles of community, and sexual harassment policies of home US institutions are operating at field sites supported by funds administered through United States institutions, awareness of these is low. Trainees sometimes work at field sites supervised by organizations not affiliated with their home institution. This intellectual cross-fostering is excellent for building collaborations and broader research networks, yet can raise additional complexities in establishing norms of behavior in the absence of an explicit, enforced policy.

Several mutually reinforcing avenues to improve the workplaces of field-based scientists are available to researchers that direct, manage, collaborate, and train at field sites and research stations. These include: raising awareness of the presence of hostile work behaviors, discrimination, harassment, and assault (particularly for women); creating guidelines for respectful behavior; and adopting independent reporting and enforcement mechanisms. The differences between the experiences of our male and female study participants also suggest that the scientific community needs to address both horizontal and vertical abuses.

These data are consistent with broader literature on workplace bullying and harassment. Many academic and corporate workplaces have zero tolerance policies for sexual harassment, but these policies are rarely attached to reporting and enforcement mechanisms that create safe spaces for victims to come forward [51], particularly as the onus is on the target of abuse to prove that the behavior is unwelcome and affects work [22]. A small minority of our survey respondents ever reported the harassment and assault they experienced, in part because very few respondents were aware of any avenue to do so. Those who had access to known reporting mechanisms may have remained hesitant to do so. Fear of reprisal was the primary reason for not reporting rape among a national study of US women [52]. Aspiring academics are exquisitely aware of the realities of finding and securing a position within small, highly specialized disciplines; as a result, targets and bystanders may be especially inhibited from reporting. Improving reporting mechanisms, however, is only a partial solution. Reporting can retraumatize the victim, precipitate retribution, and negatively affect job performance [52], [53], [54]. This may help explain why so few respondents were satisfied by the outcome of reporting harassment or assault.

Adopting principles of community, role-modeling, and embracing the collective action of support and respect [51], [55] can generate the culture change needed to prevent perpetrators from harassing and assaulting our most vulnerable colleagues – our trainees. Supervisors are the primary determinants of workplace culture [56], [57]. Therefore, principal investigators have the greatest power and responsibility to steward field sites that foster worker wellbeing and thus promote productivity and retention of junior scientists.

## Supporting Information

Materials S1 **Survey measures used for this study.**
(DOCX)
