# Supplementary material for: Survey of Academic Field Experiences (SAFE): Trainees Report Harassment and Assault
Source: PLoS One. 2014 Jul 16;9(7):e102172. doi: 10.1371/journal.pone.0102172 (PMC4100871; doi:10.1371/journal.pone.0102172)
Supplement: Materials S1 — Survey measures used for this study. (DOCX) [file pone.0102172.s001.docx]

**SUPPLEMENTARY MATERIALS S1**

The survey was conducted in two waves: the first, aimed at biological anthropologists between February 21^st^ and April 12^th^ 2013 (N=124); and the second from April 13^th^ to May 10^th^ 2013 (N=542) that allowed respondents to provide their professional discipline (page 2, question 14). This addition to the survey was in response to feedback requesting opportunities for other disciplines engaged in field research to participate in the study.

**The Biological Anthropology Field Experiences Survey**

**Page 1 of 7**

*Thank you for your interest in our survey. The Biological Anthropology Field Experiences Web Survey is designed to solicit input on the ways in which fieldwork does or does not provide a safe scholarly and research environment for all. Rather than determining the total number of instances, or percentage risk of a negative experience, our interest is in gathering stories to inform Field Directors, faculty mentors, and other researchers on the scope of the problem, and identify some of the main contributory factors to a negative environment, both to encourage improvement and to identify future areas for research.****What you will do*** *By hitting "next," you give informed consent to participate in our internet survey. Please answer these questions to the best of your ability. You can decide to end participation at any time, or not answer certain questions. We will keep your responses anonymous and your identities confidential to only the four principal investigators listed below. In any in person, in print, or on line reporting, publishing, or presenting of the data we collect from this survey all personal identifiers will be expunged. This survey and the server on which we will be storing your results are data encrypted, in order to ensure your answers are safe with us.

In order to confirm identities and make sure participants only fill out the survey once, we will ask for your email address and you will get a confirmation of completion of your survey at that address. We are interested in following up by phone with a random sub-sample of participants to hear your stories, and if you indicate you would be willing to be considered, we will additionally ask you to check a box so that we can initiate contact. You will also have the chance to check a box at the conclusion of the study to enter you into a lottery for a 1:10 chance to win a $25 Amazon gift card. You can check this box regardless of how much or how little of the survey you completed.****Risks*** *Please note that we will be asking about sexual harassment or assault experiences you may have had in the past, albeit in a general way. These questions may be triggering, and stimulation of these memories may make you uncomfortable. If you are at all worried that thinking about or sharing your experiences about sexual harassment or assault would be too mentally harmful, do not continue with this survey.****Benefits*** *We hope the results of this research will stimulate a broader conversation about mentoring, fieldwork, and support of students and peers. We believe this research has enormous benefit to the discipline, as creating a safer space for research will encourage more diverse people to pursue science, and more diverse perspectives.*

***For more information***

*This human subjects research was approved by the University of Illinois Institutional Review Board. If you have any questions about this project, please contact the primary investigator Dr. Kathryn Clancy, Assistant Professor of Anthropology at the University of Illinois, Urbana-Champaign, at 217-244-1509 or via email at*[*kclancy@illinois.edu*](mailto:kclancy@illinois.edu)*.*

*If you have any questions about your rights as a participant in this study or any concerns or complaints, please contact the University of Illinois Institutional Review Board at 217-333-2670 (collect calls will be accepted if you identify yourself as a research participant) or via email at*[*irb@illinois.edu*](mailto:irb@illinois.edu)*.*

*Again, thank you so much for your willingness to participate in our project.*

*Sincerely,

Kathryn Clancy, Assistant Professor, University of Illinois Urbana-Champaign
Katie Hinde, Assistant Professor, Harvard University
Robin Nelson, Assistant Professor, University of California Riverside
Julienne Rutherford, Assistant Professor, University of Illinois Chicago*

**Question 1**


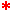
1. I agree to complete the survey

   Agree

**Page 2 of 7**

***How to fill out this survey*** *Please answer each question to the best of your ability and memory. You are free to skip over any question, or end your involvement at any time. If you wish to answer only some questions, but would like us to receive those answers, you must still click "next" through each page until you get to the "Thank You" end page.****Personal information*** *You do have to provide your email address in order to participate in this survey. However, we will use this only to create an email confirmation so that participants can only answer the survey once, and so that your identity is not impersonated by another. Participants who complete the survey (get to the Thank You page but do not necessarily answer every question) will be able to check a box to have a 1 in 10 chance of winning a gift card. We will notify you by email if you are a winner.****Follow-up*** *If you would like to be considered for a follow-up phone interview to share your stories, please be sure to check the box on the Follow-Up page. These phone interviews will be crucial to our telling the stories of participants' field experiences, and to humanize the numbers we gather in the web survey.****Personal info***

2. Valid and current email address

3. Gender

4. Sexuality

   Homosexual

   Bisexual

   Questioning

   Asexual

   Heterosexual

   Do not know

   Other

5. Race and ethnicity

   White

   White of Hispanic or Latino/a origin

   Black or African American

   Black or African American of Hispanic or Latino/a origin

   American Indian or Alaska Native

   American Indian or Alaska Native of Hispanic or Latino/a origin

   Asian

   Asian of Hispanic or Latino/a origin

   Native Hawaiian or Other Pacific Islander

   Native Hawaiian or Other Pacific Islander of Hispanic or Latino/a origin

   Multiracial/ Other race

6. Country of origin

7. Age

   18-25

   26-30

   31-35

   36-40

   41-45

   46-50

   51-55

   56-60

   61-65

   65+

8. Degree completed

   High school

   BA/BS

   MS/MA

   PhD

   Other

9. Current status

   High school student

   College student

   Graduate student

   Technician or field assistant

   Non-tenure track faculty

   Postdoctoral fellow

   Tenure-track faculty

   Tenured faculty

   Emeritus faculty

   I am not involved in academia

10. When you have done fieldwork, what kind of academic institution were you affiliated with? (check all that apply)

   High school

   SLAC

   Public 4-yr university

   Private 4-yr university

   Community college

   Research institute

   Medical school

   Other

11. Your subfield:

   Biological anthropology

   Archaeology

   Zoology

   Biology

   Geology

   Other

**Page 3 of 7**

*General climate*

*For these questions, please think about all of your experiences together as a field researcher (i.e., field site manager, researcher, research affiliate, research staff, student, etc).*

12. How many different field sites have you worked at or been a student?

   1

   2

   3

   4 or more

13. At how many field sites has the director of the site been a man?

   0

   1

   2

   3

   4 or more

14. At how many field sites has the director of the site been a woman?

   0

   1

   2

   3

   4 or more

*What were the female:male ratios of researchers at your field sites (your best guess):*

15. Site 1:

   More women than men

   About the same number of women and men

   More men than women

16. Site 2:

   More women than men

   About the same number of women and men

   More men than women

   I have only worked at one field site

17. Site 3:

   More women than men

   About the same number of women and men

   More men than women

   I have only worked at two field sites

18. Site 4:

   More women than men

   About the same number of women and men

   More men than women

   I have only worked at three field sites

19. Do any of the field sites at which you have worked or been a student had a code of conduct?

   Yes

   No

   I don't know

20. Do any of the field sites at which you have worked or been a student had an honor code?

   Yes

   No

   I don't know

21. Do any of the field sites at which you have worked or been a student had a sexual harassment policy?

   Yes

   No

   I don't know

22. From your experience, please describe the skills or attributes that make the best field director:

23. From your experience, please describe the skills or attributes that make the worst field director:

**Page 4 of 7**

*For the next set of questions, answer for your most recent, or most notable, field site for which you were not the director.*

24. I will be answering these questions about my (check one below) field site research experience.

   most recent

   most notable

25. How often did you interact with your field director compared to other field site participants?

   almost never

   rarely

   sometimes

   often

   continuously

26. How valued was your input regarding the direction of research methods and approach?

   not at all valued

   occasionally valued

   moderately valued

   very valued

   highly valued

27. How valued was your input regarding field climate?

   not at all valued

   occasionally valued

   moderately valued

   very valued

   highly valued

28. What criteria did you feel your field site director used to evaluate your performance? (check all that apply)

   Competence

   Potential

   Accomplishments

   Effort

29. Do you feel that you were held to the same standard, a higher standard, or a lower standard compared to other field site participants?

   much lower standard

   slightly lower standard

   the same as other participants

   slightly higher standard

   much higher standard

30. How gender differentiated was your field site? Examples would be that men and women do separate activities with their leisure time, or that bureaucratic or cleaning tasks get allocated disproportionately between genders.

   not differentiated

   slightly differentiated

   moderately differentiated

   very differentiated

   completely differentiated

**Page 5 of 7**

*Sexual harassment and assault*

31. With what frequency did you observe or hear about other field site researchers and colleagues making inappropriate or sexual remarks?

32. Have you ever personally experienced inappropriate or sexual remarks, comments about physical beauty, cognitive sex differences, or other jokes, at an anthropological field site? (If you have had more than one experience, the most notable to you.)

   Yes

   No

   I don't know

33. If so, who was the perpetrator?

   Someone superior in rank to me

   A peer

   Someone inferior in rank to me

   Someone unaffiliated with the field site (i.e., local resident, law enforcement, visitor)

34. What was your rank at the time of the incident?

   High school student

   College student

   Graduate student

   Technician or field assistant

   Non-tenure track faculty

   Postdoctoral fellow

   Tenure-track faculty

   Tenured faculty

   Emeritus faculty

35. Was there a mechanism for you to easily report this harassment?

   Yes

   No

   I don't know

36. If so, what was the mechanism?

37. If so, did you report it?

   Yes

   No

   I don't know

38. If you reported it, how satisfied were you with the outcome?

   very dissatisfied

   dissatisfied

   neutral

   satisfied

39. Have you ever experienced physical sexual harassment, unwanted sexual contact, or sexual contact in which you could not or did not give consent or felt it would be unsafe to fight back or not give your consent at an anthropological field site? (If you have had more than one experience, the most notable to you.)

   Yes

   No

   I don't know

40. If so, who was the perpetrator?

   Someone superior in rank to me

   A peer

   Someone inferior in rank to me

   Someone unaffiliated with the field site (i.e., local resident, law enforcement, visitor)

41. What was your rank at the time of the harassment?

   High school student

   College student

   Graduate student

   Technician or field assistant

   Non-tenure track faculty

   Postdoctoral fellow

   Tenure-track faculty

   Tenured faculty

   Emeritus faculty

42. Was there a mechanism for you to easily report this harassment?

   Yes

   No

   I don't know

43. If so, what was the mechanism?

44. If so, did you report it?

   Yes

   No

   I don't know

45. If you reported it, how satisfied were you with the outcome?

   very dissatisfied

   dissatisfied

   neutral

   satisfied

   very satisfied

**Page 6 of 7**

***Follow up***

*We would like to conduct phone interviews with a random sub-sample of participants who have experienced a chilly field climate, and/or sexual harassment or sexual assault in the field. These interviews are important because they will help us translate the quantitative data we gathered in this survey into real, human experiences that will help fellow anthropologists understand the scope of the problem, and increase their desire to find ways to address and eliminate oppression.*

*Phone interviews should take about thirty minutes. We will not share your personal information with anyone, and we will change any identifying factors that come up over the course of the interview (i.e. your name, sub-discipline, school, field site).*

46. If you would like to be considered for one of these phone interviews, please check the appropriate box (Depending on your preference, we can schedule an audio-only Skype call or a regular phone call.):

   I would like to be considered for a phone interview

   I do not want to do a phone interview

**Page 7 of 7**

Thank you very much for your participation in this survey. Please click "submit survey" below to be automatically entered into the lottery to receive a 1 in 10 chance of getting a $25 Amazon gift card, and to complete the survey.

Finally, here are some resources you may find useful:

[RAINN: Rape, Abuse & Incest National Network](http://www.rainn.org/)

RAINN’s hotlines: [Online](http://apps.rainn.org/ohl-bridge/) | Phone: 1 800 656 HOPE | [Local](http://centers.rainn.org/)

[National Sexual Violence Resource Center](http://www.nsvrc.org/)

[Feminist Majority’s collection of resources about sexual harassment](http://www.feminist.org/911/harass.html)
